# Development of a Computerized Device for Evaluating Vestibular Function in Locomotion: A New Evaluation Tool of Vestibular Hypofunction

**DOI:** 10.3389/fneur.2020.00485

**Published:** 2020-06-12

**Authors:** Po-Yin Chen, Li-Wei Chou, Ying-Chun Jheng, Shih-En Huang, Lieber Po-Hung Li, Chung-Huang Yu, Chung-Lan Kao

**Affiliations:** ^1^Department of Physical Therapy and Assistive Technology, National Yang-Ming University, Taipei, Taiwan; ^2^Department of Physical Medicine and Rehabilitation, Taipei Veterans General Hospital, Taipei, Taiwan; ^3^Department of Otolaryngology, Cheng Hsin General Hospital, Taipei, Taiwan; ^4^Faculty of Medicine and Institute of Brain Science, School of Medicine, National Yang Ming University, Taipei, Taiwan; ^5^School of Medicine, National Yang-Ming University, Taipei, Taiwan

**Keywords:** vestibular hypofunction, dizziness, vertigo, computerized functional assessment, vestibular rehabilitation, gaze and locomotion

## Abstract

To evaluate vestibular function in the clinic, current assessments are applied under static conditions, such as with the subject in a sitting or supine position. Considering the complexities of daily activities, the combination of dynamic activities, dynamic visual acuity (DVA) and postural control could produce an evaluation that better reflects vestibular function in daily activities.

**Objective:** To develop a novel sensor-based system to investigate DVA, walking trajectory, head and trunk movements and the chest-pelvis rotation ratio during forward and backward overground walking in both healthy individuals and patients with vestibular hypofunction.

**Methods:** Fifteen healthy subjects and 7 patients with bilateral vestibular hypofunction (BVH) were recruited for this study. Inertial measurement units were placed on each subject's head and torso. Each subject walked forward and backward for 5 m twice with 2 Hz head yaw. Our experiment comprised 2 stages. In stage 1, we measured forward (FW), backward (BW), and medial-lateral (MLW) walking trajectories; head and trunk movements; and the chest-pelvis rotation ratio. In stage 2, we measured standing and locomotion DVA (loDVA). Using Mann–Whitney *U*-test, we compared the abovementioned parameters between the 2 groups.

**Results:** Patients exhibited an in-phase chest/pelvis reciprocal rotation ratio only in FW. The walking trajectory deviation, calculated by normalizing the summation of medial-lateral swaying with 1/2 body height (%), was significantly larger (FW mean ± standard deviation: 20.4 ± 7.1% (median (M)/interquartile range (IQR): 19.3/14.4–25.2)in healthy vs. 43.9 ± 27. 3% (M/IQR: 36.9/21.3–56.9) in patients, *p* = 0.020)/(BW mean ± standard deviation: 19.2 ± 11.5% (M/IQR: 13.6/10.4–25.3) in healthy vs. 29.3 ± 6.4% (M/IQR: 27.7/26.5–34.4) in patients, *p* = 0.026), and the walking DVA was also significantly higher (LogMAR score in the patient group [FW LogMAR: rightDVA: mean ± standard deviation:0.127 ± 0.081 (M/IQR: 0.127/0.036–0.159) in healthy vs. 0.243 ± 0.101 (M/IQR: 0.247/0.143–0.337) in patients (*p* = 0.013) and leftDVA: 0.136 ± 0.096 (M/IQR: 0.127/0.036–0.176) in healthy vs. 0.258 ± 0.092 (M/IQR: 0.247/0.176–0.301) in patients (*p* = 0.016); BW LogMAR: rightDVA: mean ± standard deviation: 0.162 ± 0.097 (M/IQR: 0.159/0.097–0.273) in healthy vs. 0.281 ± 0.130 (M/IQR: 0.273/0.176–0.418) in patients(*p* = 0.047) and leftDVA: 0.156 ± 0.101 (M/IQR: 0.159/0.097–0.198) in healthy vs. 0.298 ± 0.153 (M/IQR: 0.2730/0.159–0.484) in patients (*p* = 0.038)].

**Conclusions:** Our sensor-based vestibular evaluation system provided a more functionally relevant assessment for the identification of BVH patients.

## Introduction

It is essential to maintain stability and clear vision when walking with head turning. Vestibular hypofunction (VHF) may cause significant walking problems. VHF typically induces vertigo (i.e., an illusion of motion) and postural imbalance due to disorders of the vestibular organs or sensory conflict (1). Hypofunction of the vestibular system can lead to various kinds of symptoms and functional problems (2). Patients with bilateral (BVH) have (1) gaze instability or oscillopsia, i.e., blurring of vision associated with head movements or during walking, (2) postural instability, and (3) gait disturbances when in darkness or the uneven ground (3, 4). Patients with BVH often need to use walking aids during locomotion such as a cane due to the increased prevalence of falls (5). In Taiwan, the overall prevalence of patients with vertigo is 3.1% (6). In terms of quality of daily life, patients with vestibular vertigo have a higher frequency of medical consultations, sick leave, and interruptions of daily activities, and the disorder results in avoidance of leaving the house (7).

Vestibular evaluation is essential to the assessment of dizzy individuals for proper treatment and further improvement of their balance and quality of life (8, 9). One previous study indicated that more than half of the participants with vestibular vertigo reported a non-vestibular diagnosis (7). For the diagnosis of VHF, appropriate assessments in cases of dizziness/vertigo are key to successful treatment and effective rehabilitation (10). Many assessments have been used in the clinical setting, such as the caloric vestibular test, computerized dynamic post-urography (CDP), the rotary chair test and the video head impulse test (11, 12). Nevertheless, in the NIH Toolbox vestibular study, some vestibular tests were excluded because of high costs, the expertise required for administration and interpretation, and/or differences in sensitivity at different age levels (13). Therefore, those assessments may not be widely utilized for quick screening or extensively applied at the community level. Most importantly, those tests are generally not administered with the patient in functional positions during dynamic tasks; instead, many tests are performed with the subject in steady and fixed positions, including in a sitting or supine position. Thus, their results may not reflect patients' real problems and difficulties in daily activities because of the lack of dynamic challenges (14).

In previous studies, head rotation with visual gaze during locomotion was considered to be a crucial function of daily life (e.g., individuals trying to cross a busy intersection or find products in a crowded market) (14–16). During dynamic locomotion with head movement, VHF patients often lose balance and clear vision (17–19), which may easily induce vertigo (4, 20). Many researchers have recommended that walking with head turning could be considered a potential way to evaluate the vital vestibular function of individuals or falling risk (14, 19, 21). Some studies have also shown that patients with vestibular disorders have deficits in gait and postural control (22–25). Postural control and trunk movement during walking are affected by the vestibular system through the vestibulospinal reflex (VSR) (26–28). The VSR enables dynamic adjustments of the trunk and lower limb muscles according to the orientation of an individual's head position in the space that is detected and built up by the integration of vestibular information with other sensory inputs. Previous studies of locomotion tests in Parkinson's disease and aging showed that dual tasks, such as rapid head turning, resulted in stiff movements or the loss of a stable rhythm in the trunk segments (15, 29, 30). However, a limited number of studies have described trunk and postural control during functional and dynamic activities. There is an imminent need for devices to assist vestibular patients and prevent fall accidents.

Currently applied assessment methods, such as the caloric test and the rotary chair test, require the test subjects to be in a supine or sitting position. Although the dynamic gait index (DGI) and the sensory organization test (SOT) contain standing or stepping tasks, they are often affected by the problem of a ceiling effect for the subjects (31–33). It is necessary to find an effective and portable tool that can evaluate dynamic visual acuity (DVA) (18) and postural control while walking (31, 34). Therefore, the purpose of this study was to (1) build a device to evaluate trunk control, trajectory, and locomotive DVA (loDVA) and (2) compare differences between healthy individuals and individuals with BVH.

## Materials and Methods

### Participants

Fifteen healthy subjects [9 males (M)/6 females (F)] and 7 patients (2 M/5 F) with VHF were recruited for this study (Table 1). Each participant signed an informed consent form that was approved by the Taipei Veterans General Hospital & National Yang-Ming University Institutional Review Board. The patients were diagnosed based on self-reported histories and the results of head thrust tests, horizontal and vertical head-shaking nystagmus tests and bithermal caloric irrigation with air (AIRSTAR, Micromedical Technologies, IL, USA) using the following criteria: bilateral vestibular hypofunction (BVH): total response of slow phase velocity <20°/s. The video head impulse tests for BVH patients were all carried out with ICS impulse video goggles (GN Otometrics, Taastrup, Denmark). Patients who wore goggles were asked to stare at a fixation dot placed on a surface 1 meter in front of them. Simultaneously, the tester standing behind the patient rotated the patient's head through a small angle (about 10–20 degrees) in a brief, abrupt and unpredictable way varying the direction and the velocity. After the test, the “gain” of the vestibular ocular reflex VOR was defined as the change in the eye angle divided by the change in the head angle during the head turn. All subjects had normal or corrected-to-normal vision with no known motor deficits or any progressive neurological disorders. They also had the ability to conduct continued head turning for at least 60 degrees to either side during walking for 5 m. BVH was diagnosed by physicians of the medical center (Table 2). BVH patients who had severely limited mobility (i.e., could not walk without the use of a walker, cane, or orthotic) and visual dysfunction were excluded from the study.

**Table 1:** Demographic data.

	**Age (years)**	**Height (cm)**	**Weight (kg)**	**BMI**
Healthy subjects (*n* = 15) (mean ± standard deviation)	34.4 ± 15.6	167.3 ± 8.2	72.0 ± 15.0	25.58 ± 4.51
Median (interquartile range) of the healthy subjects	25.0 (24.0–52.0)	169.0 (162.0–175.0)	73.0 (56.0–86.0)	25.47 (21.64–27.82)
BVH patients (*n* = 7) (mean ± standard deviation)	44.1 ± 16.0	166.2 ± 7.5	63.11 ± 6.51	22.9 ± 2.36
Median (interquartile range) of the BVH patients	46.0 (26.0–61.0)	168.0 (158.0–172.0)	64.0 (62.0–70.0)	24.22 (20.95–24.84)
Mann–Whitney U	36.500	49.500	36.000	37.000
Z	−1.131	−0.212	−1.165	−1.093
*P*-value	0.258	0.832	0.244	0.275

*Basic information of the healthy subjects and bilateral vestibular hypofunction (BVH) patients. There were no significant differences between these two groups. BMI, body mass index*.

**Table 2:** Demographic data for patients with BVH.

	**Sex**	**Diagnosis**	**Onset**	**Training**	**Caloric**	**VOR gain**
			**time**		**TR**	**L/R**
P1	F	Idiopathic BVH	2.5 m	NA	19°/s	0.64/0.60
P2	M	Idiopathic BVH	1.5 m	NA	15°/s	0.45/0.37
P3	F	Idiopathic BVH	2 m	1 m	16°/s	0.51/0.43
P4	F	Idiopathic BVH	2 m	1 m	17°/s	0.37/0.45
P5	F	Idiopathic BVH	2 m	NA	15°/s	0.26/0.32
P6	F	Idiopathic BVH	1 m	NA	17°/s	0.54/0.50
P7	M	Idiopathic BVH	1.5 m	NA	18°/s	0.58/0.53

*The onset time ranged from 1 to 3 months. BVH, bilateral vestibular hypofunction; F, female; M, male; m, month; NA, not available; Caloric TR, Total response; °/s, degree per second; VOR gain L/R, Left and right VOR gain*.

### Instrumentation

**Figure 1:**
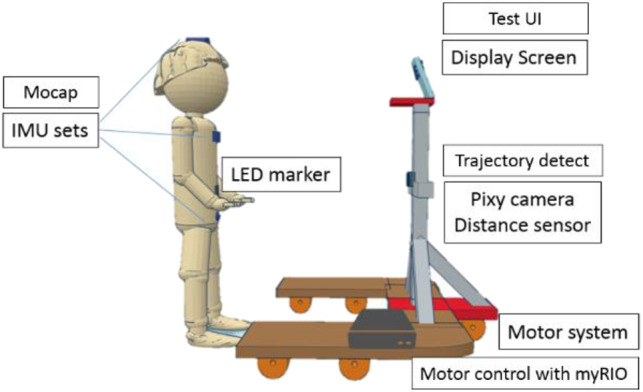
Schematic diagram of the assessment device. This evaluation tool consisted of a display screen, inertial measurement unit (IMU) motion capture (mocap), trajectory detection and motor system. There were 3 IMUs installed on segments of the subject.

A sensor-based moving platform, as shown in Figure 1, comprising 4 subsystems was established. The sensing subsystems were installed to measure the motions of the subjects and to convert those signals to interact with the control system. Furthermore, in the sensing subsystem, an inertial measurement unit (IMU) and a Pixy camera were used to observe posture and movement during the experiment. On the top of the frame of the platform, a monitor was used to implement the DVA and loDVA test.

### Sensing Subsystem

The sensing subsystem contained 3 types of sensors. An ultrasonic sensor (US-100 Ultrasonic Sensor Distance Measuring Module), with a sampling rate of 20 Hz, was used to measure the distance between the platform and the subject. One PIXY camera (CMUcam5, Austin, TX), with a sampling rate of 40 Hz and positioned close to the ultrasonic sensor, was used to measure the horizontal shift of the red LED marker fixed to the center of the user's abdomen at the same height of the camera. Through integration with the continuous distance data, we could calibrate and observe the horizontal shift (cm) of the subject in the frontal plane. Three IMUs (PNI SpacePoint SCOUT, USA) were placed, one on top of the head, one on the sternum, and one at the midpoint between the two anterior superior iliac spines and were used to measure the change in the angular motion of the head, chest and pelvis. This 9-axis IMU consists of a 3-axis accelerometer, a 3-axis gyroscope, and PNI's geomagnetic sensor suite, with a sampling rate of 125 Hz (35).

### Motor Subsystem

Stepping motors (2-phase stepping motor, PKP series) and motor drivers (2-phase, fully digital, bipolar microstepping driver), manufactured by Taiwan Oriental Motor Co., Ltd., were used to drive the platform forward and backward. With the distance data, we could program the change in speed to maintain a range of 1.5–2 m.

### Display Subsystem

A monitor was installed in the framework in front of the subject. The position of this monitor was adjusted to each subject's eye level.

### Control Subsystem

**Figure 2:**
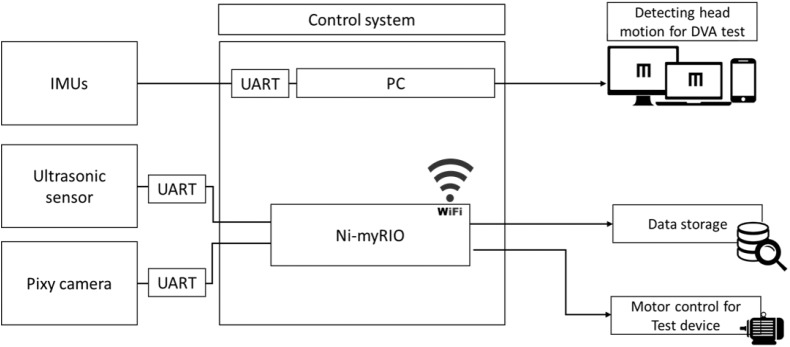
Schematic of the control system. All data from sensors were sent to the control system. Data from the inertial motion unit (IMU) were converted to angular movement and triggered changes in the display. Data from the ultrasonic sensor and video camera module (PIXY) camera were transmitted to the reconfigurable I/O (input/output, myRIO) and translated to trajectory data (cm). DVA, dynamic visual acuity and UART, universal asynchronous receiver/transmitter.

A mini PC (ASUS, Taiwan) and myRIO (National Instruments, Austin, TX) were used to receive the signals from each sensor, execute the control algorithm, transmit the control signal to the motor driver and monitor and control the motor and display subsystems (Figure 2). Using the universal serial bus to universal asynchronous receiver/transmitter (UART) module, which is a communication interface between the sensor and PC, 3 IMUs transmitted the data through cables. The PIXY camera and the ultrasonic sensor were connected to myRIO, which communicated with the PC through Wi-Fi. Additionally, the experimental data were also recorded to the mini PC.

### Control Algorithm

We used LabVIEW 2014 software (National Instruments, Austin, TX) to develop the control algorithm. The aforementioned sensing subsystems were connected to myRIO and transmitted data to LabVIEW on the mini PC for further analysis.

This control system could detect time markers from each sensing subsystem and subsequently resample the signals recorded by the sensors (IMU, 125 Hz; PIXY, 40 Hz; ultrasonic sensor, 20 Hz) to equal levels via the program in LabVIEW. When the distance between the subject and the platform was <1.5 m, the control system would increase or decrease the current motor signal by 10% to change the velocity of the motor system for acceleration or deceleration.

### Experimental Procedures

In this study, there were two experimental stages.

In the 1st stage, we measured posture and motion during walking with head motion. The subjects wore the IMU sensors and maintained a distance of 1.5 m in front of the platform while walking forward for 5 m. The subjects were asked to simultaneously focus on one image on the monitor 1.5 m in front of them. They had to perform a 2 Hz head yaw as instructed by a metronome while walking at a comfortable neck range and walking speed. After a brief rest, they walked straight backward in the same condition. Each forward walking (FW) and backward walking (BW) trial was performed twice to obtain average values. If the distance between the platform and the subject was <1.5 m during walking, a warning sign (change in the front screen color) would ask the subject to adjust their walking speed. An additional movie file shows this in more detail.

In the 2nd stage, we measured the standing DVA and loDVA. The subject was asked to take the static visual acuity (SVA) test with 55 optotypes without head motion in a standing position at the beginning of the trial. The subjects had to wear their own glasses if they need glasses to correct their vision. The subject was asked to identify the “E” letter (the letter E randomly rotated each trial by 0, 90, 180, or 270°) openings shown on the monitor 1.5 m in front of him or her. The subject then turned his or her head horizontally at a frequency of 2 Hz, in keeping with a metronome, for measurement of the standing DVA for the left and right sides. The subject could stop head rotation when answering. Before all of the DVA tests, the subject also practiced turning their head by 40 degrees (20 degrees to each size) for the DVA tests (36). After the standing DVA test and a brief rest, the subject performed the loDVA test with horizontal head rotation at 2 Hz while walking and maintaining a distance of ~1.5 m during FW and BW. In each FW or BW trial, the subjects could respond to ~3 to 6 letters while walking 5 m (see Supplementary Video 1).

### Experimental Parameters

Five variables were collected from each subject during experimental stages 1 and 2. In the 1st stage, the walking trajectory, trunk motion, and head motion were calculated. These variables are described as follows.

### Trajectory of FW and BW

**Figure 3:**
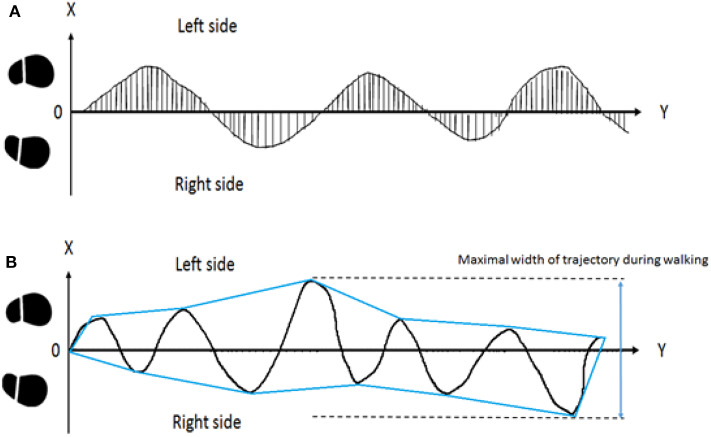
Illustration of walking trajectories. In the upper subplot **(A)**, displacement from the midline of each sample was averaged and normalized as the parameter of body stability during walking. The X displacement of the center of the body (XCoB) represents the deviation of the trajectory during FW and BW. In the lower subplot **(B)**, the enveloping line was placed over the trajectory. The maximal width from right to left was considered as sway during walking.

The trajectory recorded by the PIXY camera and the ultrasonic sensor was transformed to a 2D coordinate system. Then, the virtual straight line from the starting position of the middle center body (A) to the end target (B) was subtracted from the coordinate data. We measured the vertical distance between the trajectory and line (A)–(B) at each time stamp and obtained absolute values. Next, we averaged the values of the total data and normalized these data using half of the individual's height to represent the deviation of the walking trajectory. This method was utilized in our previous study (37). This X displacement of the center of the body (XCoB) indicates the average walking trajectory deviation of all trials. Subsequently, we also averaged the horizontal shifts of the peaks (right, RP) and valleys (left, LP) from the virtual straight line in the walking trial to obtain the swaying stability. The details of the equation are shown in equation (1) and the upper image of Figure 3.

### Maximal Width of Trajectory During Walking (Medial-Lateral Width, MLW) (cm)

We used the enveloping function to convert the trajectory data to a graphic using the enveloping line. Then, we calculated the MLW of one trial, which represented veering toward the walking direction. The increase in MLW was defined as one single or several abrupt maximal medial-lateral deviations during subjects' locomotion trials. The details are shown in the lower image of Figure 3.

Total sample number = n.

(1) $$\begin{array}{l} \text{XCoB}=\frac{\sum_{i=0}^{n}|xi|}{n} \end{array}$$

### Movement of the Head (MoH)

**Figure 4:**
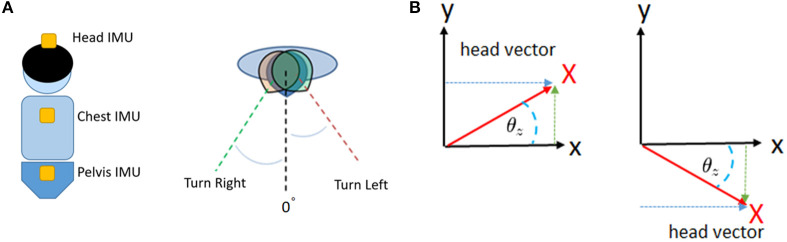
Illustration of the trunk motion analysis. Our patients wore IMUs on the head, chest, and pelvis **(A)**. We detected and recorded the yaw rotation of these body segments during walking **(B)**. After conversion, we were able to obtain continuous data regarding the degrees of rotation of the head, chest, and pelvis.

Each degree of head turning from side to side during locomotion was recorded by the IMUs, and the average and standard deviation (SD) were calculated (Figure 4). Our IMU set enabled us to measure the change in the rotation and position of the head directly. The SD of the average head rotation from left to right (right head rotation, degrees) and right to left (left head rotation, degrees) during the walking trials was considered to indicate the MoH during locomotion.

### Maximal Rotation of the Trunk (MRT) and Chest-Pelvis (CP) Ratio

**Figure 5:**
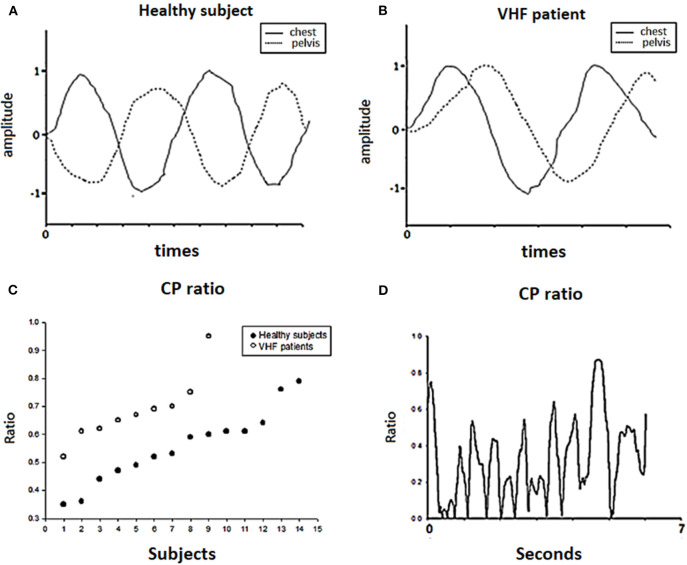
Chest-pelvis ratio (CP ratio). The angular data of the chest (C) and pelvis (P) were normalized by the maximal degrees of chest and pelvic rotation, respectively, as shown in **(A,B)**. Then, we calculated the CP ratio as the ratio of the maximal rotation in degrees and of the chest and pelvis. In **(C)**, each dot represents the chest-pelvis ratio (CP ratio) of each subject during the forward walking trial. Patients had higher CP ratio on average than healthy subjects. **(D)** Shows the change in the CP ratio among the trials for one patient.

First, we recorded the maximal rotation of the chest and pelvis and used the data to normalize the successive chest and pelvis rotation angles of each trial. The right-turning data were positive, and the left-turning data were negative. Then, we obtained the ratio by adding the chest and pelvis data and averaging out new successive data. If there was an in-phase condition between the chest and pelvis, the subject turned their chest and pelvis in the sample direction during walking, resulting in a high CP ratio. Conversely, a smaller CP ratio indicated out-of-phase cooperation of the chest and pelvis, which may be a predominant way to regulate trunk stability during walking. The details are shown in Figure 5. After recording and normalizing the degrees of chest (C) and pelvis (P) rotation, the two parameters were summed together.

(2) $$\begin{array}{l} \frac{Total\;chest\;degrees}{Maximal\;chest\;degrees}=C\Rightarrow C1,\;C2,\ldots \ldots \ldots \;Cn \end{array}$$

(3) $$\begin{array}{l} \frac{Total\;pelvic\;degrees}{Maximal\;pelvic\;degrees}=P\Rightarrow P1,\;P2,\ldots \ldots \ldots \;Pn \end{array}$$

(4) $$\begin{array}{l} CP\text{\_}ratio=C+P\;=\frac{\sum_{i=0}^{n}|Ci+Pi|}{n} \end{array}$$

### DVA Test (Standing DVA and loDVA)

The SVA and DVA tests were measured by repeatedly displaying one optotype at a time when the subject was walking and reading optotypes with head turning at 2 Hz. The patient first performed the SVA test on either side without any head movement as the baseline. Then, they performed the DVA test with head turning while standing or walking. The subject was asked to state the orientation of the “E” letters shown on the screen in front of them. Once a subject gave researchers an oral response, the researcher keyed in the answer for judgment by the program and the next trial started. In the DVA test, when the head velocity measured by the IMU on the top of head was >120 degrees/second, the system displayed the optotype “E” in a random sequence of orientations (0, 90, 180, or 270°) on the monitor for 75 ms. The monitor displayed one randomly oriented optotype 5 times for each optotype size, and the optotype size decreased in steps equivalent to a visual acuity difference of 0.1 logMAR. The 0.1 logMAR is the minimum angle resolved, in arcmin, with 1 arcmin equal to 1/60°. The converter of optotypes was equivalent to the Early Treatment Diabetic Retinopathy Study (ETDRS) visual acuity chart. After all tests, the DVA result was calculated by subtracting the correct answers in the static visual acuity test from those in the dynamic visual acuity test. The missed optotypes between the DVA and SVA were converted to the decimal visual acuity LogMAR, which comprises 11 levels ranging from 20/20 to 20/200 in vision. There were 11 lines/sizes (5 optotypes per size) in the test for each side. If the subject missed all optotypes of the same size or provided responses for all 55 optotypes in our test, they had to terminate the trial. Additional information on the visual acuity computations has been published elsewhere (38, 39). In the DVA test, the initial test size level was set to 4 levels above the smallest results in the SVA test.

### Statistics

All analyses were performed using PASW 18.0 Statistical software (SPSS, Inc., released 2009, PASW Statistics for Windows, version 18.0, Chicago). Differences in the trajectory, MLW, MoH, MRT, and CP ratio between the healthy and BVH groups were analyzed using the Mann–Whitney U test. We set the alpha level at 0.05. For standing DVA and loDVA, we also used the nonp-arametric Mann–Whitney U test for comparisons between healthy subjects and patients.

## Results

### Demographic Data

Table 1 shows the basic information of our subjects. Although the BVH patients were slightly older than the healthy subjects, there were no significant differences between the healthy subjects and BVH patients.

### Maximal Difference (i.e., MLW), Trajectory Stability (i.e., XCoB, RP, and LP), Segment Rotation (i.e., MRT), Chest-Pelvis Coordination (i.e., CP Ratio), and Movements of the Head (i.e., MoH) During FW and BW

In the motion analysis (Table 3) in stage 1, the maximal medial-lateral sway (indicated by the MLW), deviations in the trajectory of the body (indicated by the XCoB) of the trajectory of the body were significantly larger in the BVH patients than the healthy subjects during FW and BW. The variability of swaying to the right and left (indicated by the RP and LP) showed a stronger tendency in the BVH patients than in the healthy subjects; however, the standard deviation was also very large. Regarding trunk movement, the BVH patients showed greater chest and pelvis rotation (indicated by the MRT) and higher CP ratios during the FW task (Figure 5C) than did the healthy subjects. Regarding the BW task, the BVH patients showed larger degrees of maximal chest rotation compared with the healthy subjects. In terms of the MoH, the degree of rotation and the variability (SD) were similar between the VHF patients and the healthy subjects (Table 4).

**Table 3:** Control of posture and the trunk.

**FW**	MLW^*^	XCoB (%)^*^	**LP**	**RP**	Chest MRT^*^	Pelvic MRT^*^	CP ratio^*^
Healthy subjects (mean ± standard deviation)	26.5 ± 12.2	20.4 ± 7.1	10.2 ± 4.0	9.8 ± 5.2	7.3 ± 3.9	6.0 ± 2.3	0.55 ± 0.13
Median (interquartile rang) of the healthy subjects	26.3 (17.4–36.1)	19.3 (14.4–25.2)	8.4 (7.5–13.8)	8.9 (5.4–14.6)	6.9 (3.8–8.1)	5.9 (4.6–7.7)	0.56 (0.47–0.61)
BVH patients (mean ± standard deviation)	46.0 ± 6.5	43.9 ± 27.3	27.5 ± 27.9	15.4 ± 12.7	18.9 ± 7.1	16.5 ± 6.7	0.67 ± 0.14
Median (interquartile range) of the BVH patients	49.2 (28.5–65.8)	36.9 (21.3–56.9)	16.4 (6.0–43.3)	14.1 (3.4–28.1)	18.2 (14.0–25.6)	17.3 (9.3–21.9)	0.65 (0.61–0.70)
Mann-Whitney U	23.000	19.500	34.000	46.000	6.000	4.000	24.000
Z	−2.080	−2.327	−1.304	−0.458	−3.279	−3.419	−2.009
P value	0.038	0.020	0.192	0.647	0.001	0.001	0.045
**BW**	MLW^*^	XCoB (%)^*^	**LP**	**RP**	Chest MRT^*^	**Pelvic MRT**	**CP ratio**
Healthy subjects (mean ± standard deviation)	19.6 ± 9.2	19.2 ± 11.5	8.64 ± 5.0	8.14 ± 3.5	9.2 ± 4.1	7.83 ± 2.04	0.59 ± 0.11
Median (interquartile range) of the healthy subjects	19.9 (14.4–20.9)	13.6 (10.4–25.3)	7.3 (5.5–11.2)	7.1 (6.0–9.5)	8.9 (6.3–12.6)	7.6 (7.0–8.8)	0.64 (0.52–0.66)
BVH patients (mean ± standard deviation)	34.3 ± 16.9	29.3 ± 6.4	15.8 ± 11. 6	13.9 ± 10.6	15.3 ± 6.5	12.6 ± 6.1	0.68 ± 0.15
Median (interquartile range) of the BVH patients	24.9 (19.5–49.9)	27.7 (26.5–34.4)	7.3 (5.8–25.9)	10.2 (3.7–27.1)	14.8 (12.2–19.5)	13.1 (7.1–14.5)	0.64 (0.57–0.73)
Mann-Whitney U	22.000	21.000	38.000	40.000	23.000	27.000	41.000
Z	−2.150	−2.220	−1.022	−0.881	−2.079	−1.798	−0.812
P value	0.032	0.026	0.307	0.378	0.038	0.072	0.417

*FW and BW, forward and backward walking; MLW, maximal width of the trajectory during walking (medial-lateral width, MLW) (cm); XCoB, deviation of the trajectory during forward and backward walking (%) RP and LP = standard deviation of the horizontal shifts of the peaks (right) and valleys (left) (cm); MRT, maximum rotation of the trunk; CP ratio, ratio of chest and pelvis rotation; BVH, bilateral vestibular hypofunction*;

* *Statistically significant difference (P < 0.05)*.

**Table 4:** Stability of control of the head (SCH).

**FW**	**Left rotation**	**Left SD**	**Right rotation**	**Right SD**
Healthy subjects (mean ± standard deviation)	87.0 ± 24.1	8.3 ± 4.0	88.0 ± 23.2	7.3 ± 3.3
Median (interquartile range) of the healthy subjects	82.9 (63.6–104.7)	8.7 (6.0–11.2)	85.9 (66.1–103.5)	8.4 (4.9–8.7)
BVH patients (mean ± standard deviation)	76.8 ± 45.1	6.8 ± 7.2	74.9 ± 43.9	7.8 ± 5.1
Median (interquartile range) of the BVH patients	72.4 (49.9–127.6)	3.6 (2.5–9.4)	71.4 (49.1–127.4)	9.9 (2.6–12.9)
Mann-Whitney U	44.000	32.000	44.000	42.000
Z	−0.599	−1.445	−0.599	−0.740
*P*-value	0.549	0.148	0.549	0.459
**BW**	**Left rotation**	**Left SD**	**Right rotation**	**Right SD**
Healthy subjects (mean ± standard deviation)	92.7 ± 23.5	8.6 ± 4.1	93.5 ± 22.7	8.6 ± 3.8
Median (interquartile range) of the healthy subjects	83.6 (71.3–110.0)	8.6 (5.5–10.6)	87.0 (71.5–111.4)	8.2 (6.4–8.7)
BVH patients (mean ± standard deviation)	80.0 ± 46.9	5.6 ± 3.4	82.2 ± 47.5	5.6 ± 2.4
Median (interquartile range) of the BVH patients	76.5 (50.0–134.4)	4.1 (3.0–9.6)	75.6 (52.3–133.7)	4.9 (4.2–8.2)
Mann-Whitney U	45.000	29.000	46.000	25.500
Z	−0.529	−1.657	−0.458	−1.904
*P*-value	0.597	0.098	0.647	0.057

Left and right rotation = total angle of rotation to the left and right side (degree, °). SD, standard deviation; BVH, bilateral vestibular hypofunction. ^*^Statistically significant difference (P < 0.05).

### DVA During FW and BW

**Figure 6:**
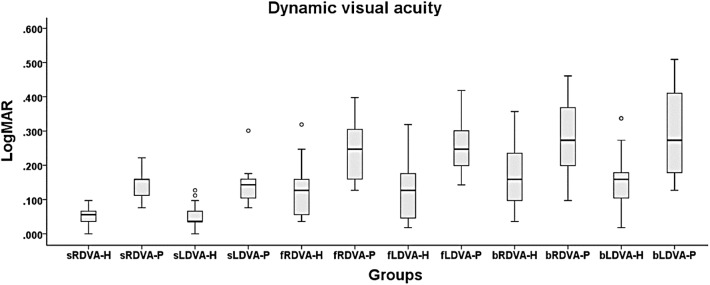
DVA during standing and walking. The 6 DVA conditions were standing and right DVA (sRDVA), standing and left DVA (sLDVA), right DVA during forward walking (fRDVA), left DVA during forward walking (fLDVA), right DVA during backward walking (bRDVA), and left DVA during backward walking (bLDVA). The unit of measurement is logMAR. During walking, the DVA was better in the healthy subjects than in the BVH patients. -H = healthy subjects and -P = BVH patients.

The healthy subjects showed better loDVA during standing and walking (Figure 6 and Table 5). The data are presented as logMAR values. Meanwhile, the DVA of the BHF patients during FW and BW was >0.2 and 0.3, which indicates a clinically meaningful reduction in visual acuity.

**Table 5:** DVA in standing and walking conditions.

	sRDVA^*^		sLDVA^*^		FRDVA^*^		FLDVA^*^		BRDVA^*^		BLDVA^*^	
	**HS**	**BVH**	**HS**	**BVH**	**HS**	**BVH**	**HS**	**BVH**	**HS**	**BVH**	**HS**	**BVH**
LogMAR (mean)	0.050	0.143	0.054	0.150	0.127	0.243	0.136	0.258	0.162	0.281	0.156	0.298
Standard deviation	0.026	0.048	0.035	0.075	0.081	0.101	0.096	0.092	0.097	0.130	0.101	0.153
Median	0.056	0.159	0.036	0.143	0.127	0.247	0.127	0.247	0.159	0.273	0.159	0.273
Interquartile range	0.036–0.076	0.097–0.159	0.036–0.076	0.097–0.176	0.036–0.159	0.143–0.337	0.036–0.176	0.176–0.301	0.097–0.273	0.176–0.418	0.097–0.198	0.159–0.484
Mann–Whitney U	3.000		7.500		17.500		18.500		24.500		23.500	
Z	−3.529		−3.211		−2.483		−2.406		−1.985		−2.071	

*The 6 DVA conditions were as follows: sRDVA, standing and right DVA; sLDVA, standing and left DVA; fRDVA, right DVA in forward walking; fLDVA, left DVA in forward walking; bRDVA, right DVA in backward walking; and bLDVA, left DVA in backward walking. The unit of measurement is the logMAR. During walking, DVA performance was better in healthy subjects (HS) than in bilateral vestibular hypofunction (BVH)*.

* *Statistically significant difference (P < 0.05)*.

## Discussion

In this study, we demonstrated that individuals with BVH had significant impairments in walking posture and gaze stability using a novel DVA device combined with motion capture. These findings may reflect the fact that BVH patients have difficulties maintaining body postural control while performing daily activities, such as watching for oncoming traffic when crossing the street and turning the head during locomotion. In addition, the problem of postural instability increases the difficulty of maintaining clear vision in patients with existing vestibular ocular reflex (VOR) instability because of vestibular dysfunction. Our study shows by using a task related to daily activities, such as walking and looking with head turning, our system may help clinical professionals develop appropriate rehabilitation programs using computerized, assistive vestibular assessments. In future clinical applications, this evaluation system may be used to monitor the progress of treatment and measure the outcome of vestibular rehabilitation. Furthermore, this evaluation may provide detailed and precise information about difficulties patients encounter in their daily lives.

### Trunk/Pelvis Postural Control (Indicated by the CP Ratio)

The most interesting observation in our study is related to postural control of the trunk. We found that patients often adopted ipsilateral turning of the chest with the pelvis, which is called “stiff motion,” during the task of walking and gazing, but healthy subjects utilized reciprocal rotation around the vertical axis of the trunk and pelvis. As shown in Table 3, the patients showed higher CP ratios indicative of this stiff strategy in both FW and BW, while the healthy subjects only used a similar type of control during BW. We could assume that they used a different postural control strategy to address the challenge in our test. Parkinson's disease patients and the elderly also use a similar strategy to control trunk interaction during locomotion. This easier but more rigid trunk motion is referred to as “en bloc” and reflects a decrease in the interactive cooperation of the chest and pelvis by turning them both in-phase or according to an orderless relationship (29). In a study of age effects on trunk control during walking, older adults often turned their chest and pelvis simultaneously, but young adults turned their chest and pelvis interactively (15). Young adults often started with chest turning and followed with pelvic turning in sequence during locomotion. In Parkinson's disease, patients tend to fix their chest and pelvis like a can during walking. Regarding the reasons for the use of the “en bloc” strategy, musculoskeletal and sensory degeneration may be the major cause in older adults, and difficulties resulting from neurological and musculoskeletal disorders or increased body imbalance may contribute to such movement in Parkinson's patients. In a study of vestibular stimulation, after 4°C caloric stimulation, Yamamoto found that during the dizzy period, healthy individuals walked with increased head and thorax yaw rotation, indicating that aberrant vestibular stimulation may cause unstable head and trunk control during walking (40). For some VHF patients, this interactive cooperation among body segments became weaker or disordered during dynamic activities (41) and improved after rehabilitation (25, 42). The stiff control of segments observed in the VHF patients in our study during walking is compatible with the findings of other studies. One previous study by Bonnier and Schilder suggests that the vestibular system not only plays a role in head control but also contributes to perceptions of the state and presence of our body in space. Vestibular patients frequently apply this strategy, which explains why muscle tightness is common in this population. In a previous 3D analysis, subjects with correct sensory input from intact vestibular organs could control their bodies using head-in-space coordination as a reference, but patients tried to control their heads according to their trunk motion (24). Therefore, patients might adopt a relatively simple method to stabilize their body instead of complex control of head and trunk stability. The cocontraction of trunk muscles could be a proper way for these patients to control and ascertain the head position. It is likely that this rigid strategy increases energy conservation more than the interactive strategy used by healthy subjects (15, 43), but rigid trunk control may provide more information regarding proprioception as a result of the increased use of muscle contraction (because of muscle feedback) and the number/frequency of steps (44, 45). In the stiff condition, proprioception could provide a proper reference from muscle activities to compensate for the weakened VSR in patients for postural control during locomotion. Even though adopting a trunk rigid when walking is easy for vestibular patients, this method can nevertheless be responded to poorly under the circumstances of more pronounced visual & somatosensory perturbations (such as walking on an irregular surface or walking with eyes closed) in BW (30).

In the BW test, the healthy subjects showed CP ratios that were similar to those of the patients, possibly due to the use of an unfamiliar motor strategy when facing an unfamiliar task. The healthy subjects and patients showed a tendency toward an increased CP ratio during BW compared with healthy subjects' results during FW. This result was in line with a previous study in which healthy subjects tended to increase their segment control based on next lower segments (en bloc) during BW rather than control segments based on the environment/space (30). In additions, both groups in this study showed less body sway from the trajectory during BW than they showed during FW, suggesting that they chose a cautious strategy to keep safe while walking (30, 43). This may explain why there were no differences between healthy subjects and patients in CP ratio during BW.

### Trunk Rotation and Body Trajectory (Indicated by the MRT, MLW, and XCoB)

Another interesting finding of our study is related to the trunk rotation and deviation in the walking trajectory: the (MRT) in the BVH patients was larger than that in the healthy subjects during locomotion. Although a previous study showed that patients with vestibular disorders tried to diminish their trunk motion to stabilize their heads (24, 34), the trunk movement in that previous study was the average degree of trunk rotation, while in our study, the parameters indicating trunk rotation were the MRT and CP ratio during walking. Compared with the healthy subjects, the patients with VHF showed a larger rotation angle (MRT), suggesting enhanced sudden movement of the chest and pelvis during the current task. The unexpectedly exaggerated trunk rotation and body deviation (indicated by the MLW and XCoB) in the VHF patients during the test could be induced by overestimation and overreaction related to abnormal vestibular function (46–48) and may consume more energy. However, there were no apparent differences in head motion between the healthy subjects and BVH patients. Therefore, the subjects and patients may have had similar head control in the current study. We speculate that this challenge of the task might not be difficult enough to affect head motion for BVH patients but may be adequate to distract them from complex postural control during walking. In addition, larger walking trajectory deviations and medial-lateral sway distances during FW and BW also revealed deficits in gait and balance control. The result of a larger deviation during walking with head movement echoes our previous study findings (37). One previous study suggested that abnormal backward walking is a strong indicator for patients with dizziness (43). In our study using more intense stimulation to the vestibular system (walking backward with 2 Hz head yaw), we found a significantly greater average deviation of walking trajectories and medial-lateral sway in both FW and BW in patients compared with healthy individuals.

### Gaze Instability and loDVA

In the DVA test, the results show a tendency toward DVA deterioration with increasing task complexity. According to previous studies, LogMAR > 0.2 or 0.3 determined by the DVA test using the “E” test, is considered abnormal (49, 50). This indicates that our system provides the ability to differentiate DVA between healthy and VHF groups during dynamic tasks under the circumstances of real daily activities. In previous studies, researchers modified the ETDRS chart, which was designed for visual tests of the clinical implication of the DVA in the sitting position (13, 51). The performance can be converted to logMAR. If the positive logMAR difference compared with the SVA is >0.2 to 0.3 during dynamic head rotation, the result will be considered a DVA deficit. In the current results, we noted that the subjects may have more blurred vision during locomotion and head turning than in the standing position. The reasons might be that the head is unstable due to body sway or stepping of the lower limbs. Thus, the VOR adjustment from abnormal vestibular information worsened the gaze acuity. This result is similar to that of DVA tests on the treadmill or in simulated human locomotion. More importantly, in contrast to tests in previous studies, we provide a new method allowing the real sensation of locomotion acceleration, closely reflecting the reality of the daily life of the subjects (17, 18).

The tendency toward higher logMAR scores for loDVA during BW may echo the findings of studies performed with the head fixed (52). The researchers found that fixing the head relative to the trunk aggravated gaze instability rather than providing relief during walking. There are two possible explanations for our results. One is that the multiple functional tests may distract patients from concentrating on visual tasks. The subjects need to turn their heads, keep walking, and pay attention to the visual target, which seems to be common for healthy subjects in daily life during forward locomotion. However, patients may be scrambling to react to the complex or unfamiliar circumstances (53–55). Another explanation is that rigid body control makes it difficult to dampen the vibration of stepping and control head stability (52, 56). Therefore, patients may try to fix their heads relative to their trunks instead of in space, producing greater sway intensity, which transfers from the lower body to the head.

### Advantages of loDVA

With the loDVA test, we could use dynamic tasks during standing and locomotion to reflect problems or symptoms that may not be easily detected in the supine or sitting position used in current evaluations. In addition, humans face multiple challenging tasks in daily activities, and our platform mimics some functionally relevant activities during walking. Therefore, this platform may be beneficial to researchers for the in-depth investigation of daily functions related to BVH. Regarding the loDVA score, according to a previous study (57), the authors indicated that at different speeds, the subjects would adapt relative strategies to integrate gaze. Although the subjects could not walk quickly in our study, it is difficult to exclude the effect of head pitch or yaw rotation and the interaction of the angular and linear VOR. Therefore, loDVA might reflect the composite performance of vestibular function.

### Limitations and Future Work

First, the number of VHF subjects was small. Second, the feeling of restraint by cables or belts used to fix the sensors may have made some participants uncomfortable. The results of our study highlight the need for future research on wireless IMUs and customized vests for subjects. In future studies, we will replace the original motor with a noise-canceling motor and recruit more subjects to validate the power of our novel system for community use.

## Conclusion

Our novel platform highlights greater trunk and body sway and walking trajectory deviations and reduced DVA during locomotion, especially in BVH patients. BVH patients adopt a stiff model for trunk movement, using more proprioception as a reference for head stabilization. Our real-life, an overground walking system provides measurements that are more objective than those obtained by conventional evaluation scales. Features of this platform such as portability, the speed of the preparation (7–8 min), and small size of our sensors and motored platform also may provide advantages over an ocular motion capture system. In the clinic and communities, it may expand the feasibility of gait analyses and provide more functionally relevant outcomes for rehabilitation.

In future studies, we will recruit UVH patients and compare the results of both patient sets with those of current, well-designed assessments or scales in the clinic for the establishment of a proper evaluation protocol and determination of the ideal cutoff for balance risk.

## Data Availability Statement

The datasets generated for this study will not be made publicly available. The data generated during the current study are not publicly available due to protection of individual privacy in the ethics approval and consent but are available from the corresponding author on reasonable request.

## Ethics Statement

The studies involving human participants were reviewed and approved by Institutional Review Board, Taipei Veterans General Hospital (2018–05-012C) Tel: +88622875–7384 Email: irbopinion@vghtpe.gov.tw and Institutional Review Board, National Yang-Ming University (YM104086F) Tel: +88622823–9753 Email: irb@ym.edu.tw. The patients/participants provided their written informed consent to participate in this study.

## Author Contributions

P-YC: study design, the development of the software and hardware, and data analysis and manuscript writing. L-WC: study design. Y-CJ and S-EH: the development of the software and hardware and data collection and analysis. LP-HL: subject diagnosis and evaluation. C-HY: study design, the development of the software and hardware. C-LK: study design, subject diagnosis and evaluation, and manuscript writing.

## Conflict of Interest

The authors declare that the research was conducted in the absence of any commercial or financial relationships that could be construed as a potential conflict of interest.

## Supplementary Material

Supplementary Video 1 DEMO movie.

## Abbreviations

VHF: vestibular hypofunction
DVA: dynamic visual acuity
FW: forward
BW: backward
M: median
IQR: interquartile range
BVH: bilateral vestibular hypofunction
medial-lateral width, MLW (cm): maximal width of the trajectory during walking
loDVA: locomotion DVA
CDP: computerized dynamic posturography
VSR: vestibulospinal reflex
DGI: dynamic gait index
SOT: sensory organization test
M: male
F, female IMU: inertial measurement unit
mocap: motion capture
PIXY: video camera module
UART: universal asynchronous receiver/transmitter
RIO: reconfigurable I/O
SVA: static visual acuity
XCoB: X displacement of the center of the body
right: RP the horizontal shifts of the peaks
left, LP: valleys
MoH: movement of the head
SD: standard deviation
MRT: maximal rotation of the trunk
(CP) ratio: chest-pelvis
(C): chest
(P): pelvis
logMAR: logarithm of the minimum angle of resolution
sRDVA: standing and right DVA
sLDVA: standing and left DVA
fRDVA: right DVA during forward walking
fLDVA: left DVA during forward walking
bRDVA: right DVA during backward walking
bLDVA: left DVA during backward walking
VOR: vestibular ocular reflex.
